# Trends in bacteriological spectrum and antibiotic susceptibility on blood culture in pediatric cardiac patients at a tertiary childcare health facility

**DOI:** 10.12669/pjms.38.5.5072

**Published:** 2022

**Authors:** Mudasser Adnan, Muhammad Sohail Arshad, Hafiz Anwar-ul-Haq, Hashim Raza

**Affiliations:** 1 Mudasser Adnan, FCPS (Paeds Medicine) Department of Pediatric Cardiology, The Children’s Hospital & The Institute of Child Health, Multan, Pakistan; 2 Muhammad Sohail Arshad, FCPS (Paeds Cardiology) Department of Pediatric Cardiology, The Children’s Hospital & The Institute of Child Health, Multan, Pakistan; 3 Hafiz Anwar-ul-Haq, FCPS (Paeds Medicine) Department of Pediatric Cardiology, The Children’s Hospital & The Institute of Child Health, Multan, Pakistan; 4 Hashim Raza, FCPS (Paeds Medicine) Department of Pediatric Nephrology, The Children’s Hospital & The Institute of Child Health, Multan, Pakistan

**Keywords:** Blood culture, *Salmonella typhi*, Acinetobacter baumannii

## Abstract

**Objectives:**

To report trends in bacteriological spectrum and antibiotic susceptibility on blood culture in admitted pediatric cardiac patients at a Tertiary Childcare Health Facility.

**Methods:**

This cross-sectional observational study was conducted at the Department of Pediatric Cardiology, The Children’s Hospital and Institute of Child Health, Multan from January 2018 to December 2020. We included admitted children of both genders aged one day to 12 years and whose blood sample was sent for blood culture analysis. Gram staining was used to identify isolated organisms. Distribution of types of strains, bacterial isolates and antimicrobial sensitivity/resistance were recorded.

**Results:**

During the study period, a total of 772 blood samples were sent for blood culture analysis, out of which, 154 (19.9%) turned out to be positive. Mean age was noted to be 1.12±2.3 years. Gram negative rods were the most frequently noted strains found among 69 (44.8%) cases. A total of 131 strains were found to have bacterial isolates. *Salmonella typhi* was the commonest bacterial agent noted in 30 (19.4%) cases while Coagulase Negative Staphylococcus in 18 (11.7%) and acinetobacter baumannii in 16 (10.4%).

**Conclusion:**

Blood culture positivity rate was found to be 19.9%. Gram negative rods were the most frequently noted strains. *Salmonella typhi*, Coagulase Negative Staphylococcus and Acinetobacter baumannii were found to be the commonest bacterial isolates responsible. Routinely used antibiotics like Ciprofloxacin, Cefotaxime, Ceftizadime and Ampicillin were found to have high rates of resistance against most commonly found bacterial isolates.

## INTRODUCTION

Multi-drug resistant (MDR) infections are on the rise globally and cause major burden in terms of morbidity and mortality in pediatric age groups.^1^,^2^ Blood stream infections are considered to result in grave consequences causing death among 3-18% children.^3^,^4^ Additionally, emotional and financial impact is thought to be huge especially among developing countries.^5^,^6^ Among children, major risk factors for infections are immature innate and adaptive immunity that is further affected in the presence of infections, and congenital heart diseases. Regional data suggests prevalence of blood culture positivity among admitted children to be between 7-86%.^7^,^8^

Present knowledge about the prevalence and patterns of causative agents responsible for infection and its antimicrobial sensitivities are very important aiming management of blood-stream infections among admitted children. No local data exists exhibiting patterns of microorganisms and their antimicrobial susceptibility from any cardiac childcare health facilities. Traditionally, gram negative bacteria form major proportions of causative agents found among children admitted for various kinds of cardiac issues. Current study aimed at finding trends in bacteriological spectrum and antibiotic susceptibility on blood culture in admitted pediatric cardiac patients at a tertiary childcare health facility of South Punjab, Pakistan.

## METHODS

This study was conducted at the Department of Pediatric Cardiology, The Children’s Hospital and Institute of Child Health, Multan from January 2018 to December 2020. Approval from Institutional Ethical Committee was taken (Ref# 404/20, dated:19-02-2020). Written consent was sought from parents/guardians of all study participants.

### Inclusion Criteria:

We included admitted children of both genders aged one day to 12 years and whose blood samples were sent for blood culture.

### Clinical Criteria:

Clinical criteria for requesting blood culture included any of the following: fever as temperature above 37.5°C or temperature below 36.5 °C

### Laboratory Criteria:

Laboratory criteria for requesting blood culture included any of the following: Leukocyte count < 4000/mm^3^ or >10000/mm^3^, CRP above 10mg/L, bradycardia or tachycardia, tachypnea, infiltrates on chest X-ray, turbid urine, dysuria, thrombophlebitis, abdominal pain or tenderness.^9^

### Exclusion Criteria:

Children receiving any kinds of oral or parenteral antibiotics in last three days were excluded. Children whose parents/guardians did not allow being part of this study were also not enrolled.

### Data Collection:

Five ml blood sample was acquired adopting strict aseptic conditions prior to initiating any kind of antibiotic treatment. All blood samples for this study were sent to central institutional laboratory aiming culture and sensitivity assessment. All blood cultures were done using standard bottle for inoculation and incubation period of five days. Gram staining was used to identify isolated organisms. Blood culture and sensitivity assessment by institutional laboratory was done as per standard protocol. Identified microorganisms were confronted to most frequently utilized antimicrobials for susceptibility pattern adopting “Kirby Baur Disc Diffusion” technique. All study information was recorded on specifically designed proforma for this study.

### Statistical Analysis:

Qualitative variables like gender, distribution of types of strains, bacterial isolates and antimicrobial sensitivity/resistance were highlighted as frequency and percentages. Age was represented as mean and standard deviation. SPSS version 26.0 was used for data analysis.

## RESULTS

During the study period, a total of 772 blood samples were sent for blood culture analysis, out of which, 154 (19.9%) turned out to be positive. Among those 154 cases, there were 95 (61.6%) male and 59 (38.4%) female. Mean age was noted to be 1.12±2.3 years ranging between day-1 to 12 years. Overall, mean body weight was noted to be 7.1±3.6 kg. There were 562 (72.8%) children aged below one year, 108 (14.0%) between one to five years, 52 (6.7%) between 5 to 10 years while remaining 50 (6.5%) were aged between 10 to 12 years.

Distribution of types of strains found are shown in Table-I while gram negative rods were the most frequently noted strains found among 69 (44.8%) followed by gram positive cocci in 43 (27.9%).

**Table I:** Distribution of Types of Stains Found (n=154).

Types of Stains	Number (%)
Gram Positive Rods	2 (1.3%)
Gram Positive Cocci	43 (27.9%)
Gram Negative Rods	69 (44.8%)
Gram Negative Cocci	17 (11.0%)
Yeast Cells	23 (14.9%)

 A total of 131 strains were found to have bacterial isolates. *Salmonella typhi* was the commonest bacterial agent noted in 30 (19.5%) cases while Coagulase Negative Staphylococcus in 18 (11.7%) and acinetobacter baumannii in 16 (10.4%). Complete frequencies of different bacterial isolates with respect to age distribution noted in the present study is shown in Table-II.

**Table II:** Frequency and Distribution of Bacterial Isolates with respect to Age Groups (n=131).

Types of Bacterial Isolates	Number (%)	Age Groups (years)			

		<1	1-5	5-10	>10
*Salmonella Typhi*	30 (22.9%)	22	3	2	3
Acinetobacter Baumannii	16 (12.2%)	12	2	1	1
Coagulase Negative Staphylococcus	18 (13.7%)	13	2	2	1
Staphylococcus Epidermidis	16 (12.2%)	12	1	2	1
Serratia Marcescens	12 (9.1%)	10	1	-	1
Klebsiella Pneumoniae	6 (4.6%)	4	1	-	1
Burkholderia SPP	5 (3.8%)	3	2	-	-
Enterobacter Cloacae	4 (3.1%)	3	1	-	-
Enterococcus Faecalis	3 (2.3%)	2	-	1	-
Acenetobacter Lwoffii	2 (1.5%)	1			1
Bacillus SPP	2 (1.5%)	2			-
Eschericia Coli	2 (1.5%)	1			1
Methicillin Resistant Staphylococcus Aureus	2 (1.5%)	1		1	-
Pseudomonas Aeruginosa	2 (1.5%)	1		1	-
Pseudomonas Luteola	2 (1.5%)	-	1	1	-
Serratia Liquefaciens	2 (1.5%)	1	1	-
Others	7 (5.3%)	1	3	2	1

Total	131 (100%)	89	18	13	11

Cefepime (90.0%) and cefuroxime were noted to have the highest sensitivities against *Salmonella typhi* while chloramphenicol (80.0%) and con-trimoxazole (66.7%) were found to be having highest resistance patterns. Acinetobacter Baumannii were 100% sensitive to Tigecyclin, Colistin and Chloramphenicol. Coagulase negative staphylococcus were 100% resistant to azithromycin and had 83.3% resistance for oxacillin. Amikacin and Teicoplanin showed 100% sensitivity to Coagulase negative staphylococcus. Sensitivity and resistance patterns of most frequent bacterial isolates found in the present study are shown in Table-III.

**Table III:** Antimicrobial Sensitivity and Resistance Patterns against Most Frequent Bacterial Isolates.

Bacterial Isolate	Most Sensitive Antibiotic	Sensitivity (%)	Most Resistant Antibiotic	Resistance (%)
*Salmonella Typhi* (n=30)	Meropenem	93.3	Chloramphenicol	80.0
	Imipenem	86.7	Ciprofloxacin	80.0
	Cefepime	70.0	Co-trimoxazole	66.7
	Ceftriaxone	66.7	Ampicillin	50.0
Acinetobacter Baumannii (n=16)	Tigecyclin	100	Clindamycin	100
	Colistin	100	Cefepime	81.8
	Chloramphenicol	100	Piperacillin Tazobactam	81.3
	Tobramycin	90	Ceftazidime	81.3
Coagulase Negative Staphylococcus (n=18)	Amikacin	100	Azithromycin	100
	Teicoplanin	100	Oxacillin	83.3
	Vancomycin	100	Tigecyclin	69.2
	Doxycyclin	87.5	Ciprofloxacin	50
Staphylococcus Epidermidis (n=16)	Amikacin	100	Ampicillin	100
	Vancomycin	100	Amoxicillin	100
	Linezolid	93.8	Co-Amoxiclav	87.5
	Doxycycline	77.8	Cefepime	87.5
Serratia Marcescens (n=12)	Polymixin	100	Co-Amoxiclave	100
	Fosfomycin	100	Piperacillin Tazobactam	91.7
	Amikacin	58.3	Cefotaxime	91.7
	Clindamycin	55.6	Doxycyclin	83.3
Klebsiella Pneumoniae (n=6)	Tigecyclin	100	Clindamycin	100
	Polymixin	100	Co-Amoxiclav	83.3
	Colistin	100	Cefotaxime	80
	Chloramphenicol	100	Doxycyclin	80

## DISCUSSION

Present study is the 1^st^ study from Pakistan aimed at finding trends in bacteriological spectrum and antibiotic susceptibility on blood culture in admitted pediatric cardiac patients at a tertiary childcare health facility. Positive culture rate of 19.9% was seen in the present study. Regional data from Saudi Arabia evaluating bloodstream infections among children admitted to undergo cardiac surgery showed blood culture positivity rates of 8.6% which is lower than what we noted in the present study.^10^ In the past, studies conducted at different pediatric cardiac healthcare facilities have found culture positivity rates between 6-8%^2^,^11^,^12^ while lack of local data hinders showing positive blood culture rates among admitted patients at pediatric cardiology units. Local general data among children have found blood culture positivity rates of 24%^13^ while data from Palestine noted positive blood culture rates to be 13.2%.^14^ Data from India shows large variation exhibiting positive culture rates between 7-89%.^8^ This variation between positive blood culture rates could be attributed to different etiological agents, past history of antibiotic usage and difference in blood culture analysis methods at different settings.^15^

In the present study, distribution of types of strains found gram negative rods to be the most frequently noted strains found among 69 (44.8%) followed by gram positive in 43 (27.9%) children. A total of 131 strains were found to have bacterial isolates. *Salmonella typhi* was the commonest bacterial agent noted in 30 (22.9%) cases while Coagulase Negative Staphylococcus in 18 (13.7%) and acinetobacter baumannii in 16 (12.2%). *Salmonella typhi* showed resistance against some of the most frequently used empirical antibiotics like ciprofloxacin (80.0%) in the present study while good sensitivity was found against meropenem 93.3%, imipenem 86.7% and Cefepime 70.0%. Local studies have stated high resistance rates of salmonella types against some of the most commonly used antibiotics like ciprofloxacin, levofloxacin and Ofloxacin.^16^ In the past, multi-drug resistance (MDR) against salmonella isolates have been found to be around 20%^17^ while data from India has showed a significant decrease exhibiting current MDR rates of 4.7%.^18^ Recent data from South Punjab Pakistan has shown high rates of resistance shown by *salmonella typhi* against Oxytetracycline (70.1%) while Ofloxacin (90.4%) and Cefepime (89.7%) had the highest sensitivity.^19^

We found Acinetobacter Baumannii to have 100% sensitivity against Tigecyclin, Colistin and Chloramphenicol while Clindamycin (100%) and Cefepime (81.8%) were having highest resistance rates. A local study from Karachi analyzing 100 Acinetobacter isolates from neonatal intensive care unit noted these to be completely resistant (100%) to most commonly used antibiotics like Cefipime, gentamicin, ceftazidime and piperacillin-tazobactam.^20^ Increasing rates of resistance against most commonly used antibiotics is pointing towards a direction where we should be using antibiotics more judicially following the right dosage schedules and duration while local guidelines needs to be revised regarding the use of most commonly adopted empirical antibiotic therapies.

### Limitations of the study:

As this was a study conducted at a single center, our findings cannot be generalized regarding etiological agents and antibiotic sensitivities. Overall sample size was not very big so further studies involving multiple centers and different sets of patients are needed to further verify the findings of this study. We could not differentiate cases referred from different units of our hospital or those who were referred from other healthcare facilities. We were also unable to relate linkage between commonly found isolates and site of infection which would have given further insight. We could not estimate effects of malnutrition in the blood culture positive patients as well.

## CONCLUSION

Blood culture positivity rate was found to be 19.9%. Gram negative rods were the most frequently noted strains. *Salmonella typhi*, Coagulase Negative Staphylococcus and Acinetobacter baumannii were found to be the commonest bacterial isolates responsible. Routinely used antibiotics like Cefotaxime, Ceftizadime and Ampicillin were found to have high rates of resistance against most commonly found bacterial isolates.
